# Electronic Relaxation Dynamics of 6‑Azauracil: The Effect of Ring Substitution on Intersystem Crossing

**DOI:** 10.1021/acs.jpca.5c04895

**Published:** 2025-09-25

**Authors:** Moti Raj Chudali, Susanne Ullrich

**Affiliations:** Department of Physics and Astronomy, 66650 University of Georgia, Athens, Georgia 30602, United States

## Abstract

In an effort to obtain a fundamental understanding of substitution effects on the photodynamic response of the canonical nucleobases, recent spectroscopic studies have focused on derivatives with single atom substitutions. Uracil undergoes ultrafast internal conversion to the ground state facilitated by various ring distortions at the ethylenic bond. In 6-azauracil, site-specific nitrogen substitution at this carbon double-bond restricts access to ethylenic conical intersections that lead back to the ground state. Instead, intersystem crossing into the triplet manifold becomes highly efficient. This study uses time-resolved photoelectron spectroscopy to investigate the photodynamics of 6-azauracil with particular focus on the role of the lowest singlet excited state and hindrance of ethylenic deactivation coordinates in promoting intersystem crossing.

## Graphical abstract

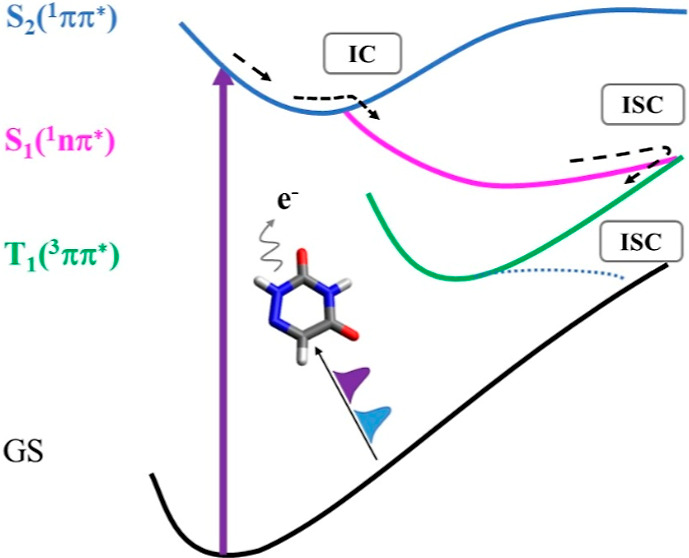

## 1 Introduction

DNA and RNA nucleobases and their subunits are good absorbers of ultraviolet (UV) radiation, particularly at wavelengths below 300 nm. When photoexcited by highly energetic UVC light, the absorbed photon energy is sufficient to cause bond dissociations and molecular fragmentation. However, the canonical nucleobases possess inherent photoprotective properties that preserve the structural integrity of the molecule by dissipating the excitation energy into heat. Previous studies on the nucleobases, such as time-resolved spectroscopic experiments and quantum chemical calculations,^1–14^ showed that the dominant relaxation mechanism to the ground state is radiationless. This process involves ultrafast internal conversion (IC) from the bright state to lower singlet excited states and the ground state. These pathways effectively convert electronic into vibrational energy. Slower intersystem crossing (ISC) and triplet state dynamics gain relevance on longer time scales.^15–19^ In contrast, substituted nucleobases have distinct photophysical properties compared to the canonical nucleobases.^20–26^ For example, in thiobases—formed by exocyclic substitution of an oxygen atom with sulfur—the primary mode of relaxation is ISC with quantum yields reaching unity.^27–39^ Thionation introduces low lying excited states characterized by sulfur localized orbital transitions that are red-shifted. The switch in deactivation mechanism arises from a selective lowering of the singlet excited state minima, while energies of conical intersections (CIs) remain mostly unaffected. This introduces significant energy barriers that make CIs with the ground state inaccessible. Instead, the excited state population is funneled into the triplet manifold via the lowest singlet state, S_1_, of ^1^nπ* orbital character. Furthermore, the lifetimes of the involved triplet states are sensitive to the position and degree of the substitution.^22,29^ Due to these properties, thiobases have received significant attention for potential applications that are mediated by triplet states. For example, the therapeutic efficacy of photosensitizers in cancer treatments relies on long-lived triplet states for reactive singlet oxygen generation.^27,40–43^ The significance of long-lived triplet states extends beyond biomedical applications, with implications across a variety of photonic and energy related technologies. Various devices can benefit from long-lived triplet states, however, managing and effectively using these states is a challenge as they can also quench the desired functions.^44,45^ Therefore, significant research is going into the fundamental understanding of triplet state processes and their utilization to improve device efficiency and performance. For example, processes involving triplet states such as triplet–triplet energy transfer and triplet–triplet annihilation are under active investigation for potential roles in emerging technologies for photovoltaics, photocatalysis, and photonic materials.^46–50^ In that respect, thiobases may provide new opportunities in the design of triplet active materials. Conversely, fluorescent analogues of the nucleobases can serve as effective biosensors for light-based detection of biological processes.^51,52^ These compounds minimize ISC and instead exhibit efficient IC to the lowest singlet excited state, which enhances radiative decay pathways such as fluorescence. This poses the question of whether other types of single-atom substitutions can similarly tune the photoproperties of the nucleobases, in particular their triplet yields and lifetimes, toward specific applications. In this context, azauracils have been investigated for a number of biological effects, including antitumor and anticancer properties as well as inhibitors of certain enzymes or microorganisms.^53–59^

Aza analogs are one example of a class of modified nucleobases in which a ring carbon atom is replaced with nitrogen. They are good candidates for a series of systematic studies on how the type and position of a substituent can control the nucleobase photodynamics and as such of interest to both fundamental and application-oriented studies. In fact, earlier work by Kobayashi et al.^60^ examined the excited state properties of aza analogs of the nucleobases in acetonitrile solution and classified them into two categories based on their tendencies for either ISC or IC. It was found that 6-azauracil (6AU) as well as 8-azaadenine (8AA) exhibit efficient ISC, whereas 5-azacytosine (5AC) and 8-azaguanine (8AG) predominantly undergo IC to the ground state. This categorization is attributed to the presence or absence of a low-lying singlet ^1^nπ* state to promote ISC. Newer theoretical studies, however, have questioned this broad generalization. Although the lowest singlet ^1^nπ* state plays a significant role in ISC in substituted nucleobases and is frequently referred to as the doorway to the triplet manifold, it is not necessarily the main reason for the distinctly different behaviors. One example is 5AC, where a ^1^nπ*/^3^ππ* crossing exists but ISC is less efficient than in 6AU due to lower spin–orbit coupling (SOC), a larger energy gap in the degenerate region, and competition with efficient IC to the ground state.^61^

6AU, which is formed by substitution at the C_6_ position of uracil, is the focus of the present work (see Figure 1). The fundamental and intrinsic photophysical properties of 6AU are not yet entirely understood. The lack of gas-phase spectroscopic studies that investigate the molecule under isolated conditions prevents a direct comparison to the solution-phase behavior of photoexcited 6AU and also to its canonical counterpart uracil. Both comparisons are particularly informative to distinguish environmental effects from intrinsic properties and to understand the mechanistic details governing the 6AU photodynamics. According to the generally accepted photophysical model for uracil, the singlet excited states connect to the ground state through several distinct ethylenic CIs and the C5=C6 bond plays an important role in the ultrafast relaxation, while ring opening and planar pathways are less significant.^13,32,62–64^ Consequently, a single-atom substitution at this position modifies the electronic properties and rigidity of an active bond and these changes can potentially have drastic effects on the deactivation mechanism. The substitution with a N atom at the C6 position in uracil introduces a lone pair of electrons and forms a N6–N1 single bond as well as a C5=N6 double bond in the ring.^65,66^ When comparing the optimized geometrical structure of uracil and 6AU, the C5=N6 bond in 6AU is shorter than the C5=C6 bond in uracil. This can be explained by the smaller size and higher electronegativity of the N atom which attracts bonding electrons and enhances orbital overlap.^65^ Aza-substitution increases the rigidity of the C5=N6 double bond compared to C5=C6 because the lone pair of electrons on the N6 atom enhances the resonance of the heterocyclic ring.^65,66^ Furthermore, the N6 lone pair contributes electron density to orbitals that are active in the electronic transitions and alters the electronic character of the excited states in 6AU compared to uracil. Combined, the electronic and geometric effects of aza-substitution lead to significant changes in the topologies of the excited state surfaces and, from a theoretical perspective, distinct photodynamics from uracil are to be expected.

**Figure 1:**
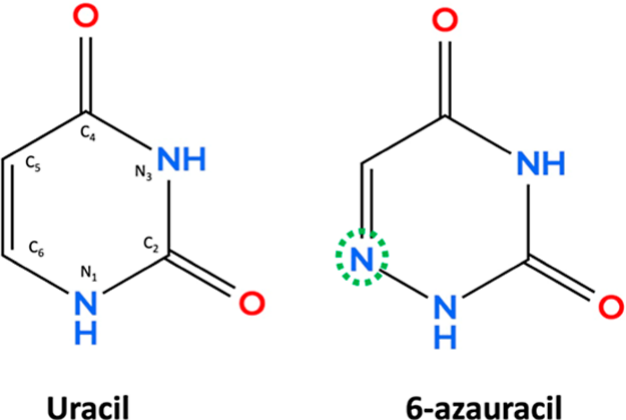
Chemical structures of uracil and 6-azauracil, in which the N atom substitution at the C6 position is highlighted by a green circle.

Transient absorption measurements of 6AU in acetonitrile by Kobayashi et al.^67^ revealed a high quantum yield for ISC as well as high singlet oxygen generation, while IC back to the ground state was negligible. According to their proposed photophysical model, two factors may be responsible for the distinctly different behavior from uracil. First, aza-substitution introduces a N atom which contributes to the formation of additional nπ* states, that are key for efficient ISC. Furthermore, the rigidity of the C5=N6 double bond in the ring hinders direct IC via ethylenic funnels to the ground state and instead favors ISC. Therefore, singlet to triplet ISC becomes the dominant relaxation pathway and only a minor fraction of the excited state population internally converts to the ground state.^67^ Transient absorption measurements with a pump pulse at 264 nm, for example, indicated a three step sequential decay with time constants <0.3 ps, 5.2 ps, and >1000 ps, which are attributed to the population decay of the S_2_(^1^ππ*), S_1_(^1^nπ*)_,_ and T_1_(^3^ππ*) states, respectively.^68^ In addition to this, similar measurements with nanosecond time resolution, have determined the triplet state lifetime to be approximately 190 ns.^67^ The involvement of the singlet S_1_(^1^nπ*) excited state is further supported by emission studies. Photoexcitation at 248 nm to the bright S_2_(^1^ππ*) does not yield any detectable fluorescence while direct excitation with 308 nm to the mostly dark S_1_(^1^nπ*) shows weak fluorescence at around 420 nm with a quantum yield of (4.2 ± 0.4)×10^–3^.^67,69^ The fluorescence lifetime of less than 30 ns indicates that emission originates from the lowest singlet state, S_1_(^1^nπ*), minimum as opposed to phosphorescence from a triplet state.

Several ab initio calculations and dynamics simulations have been performed in an attempt to explain the photodynamics of 6AU. A static picture of the photophysical properties of 6AU is gleaned from independent ab initio quantum chemical calculations by Marian and Etinski^66^ (RI-CC2) and Borin et al.^65^ (CASSCF/CASPT2). Both propose two possible relaxation routes: one S_2_(^1^ππ*) → S_1_(^1^nπ*) → T_1_(^3^ππ*) and the other S_2_(^1^ππ*) → T_2_(^3^nπ*) → T_1_(^3^ππ*) but they favor the first. Marian et al. reason that multiple S_2_/S_1_ CIs efficiently populate the S_1_(^1^nπ*), which then acts as a doorway to the triplet manifold and the T_1_(^3^ππ*) minimum, although, SOC for ISC processes in 6AU are similarly low as in U. In contrast, Borin et al. report a sizable SOC between the S_1_ and T_1_ which enhance ISC in 6AU. Furthermore, S_2_/S_1_ and S_1_/T_1_ crossings occur near the minimum and are almost barrier-free whereas direct IC from S_2_ or S_1_ to the ground state is effectively blocked by unsurpassable barriers. Molecular dynamics simulations by Nanbu and Iwasa^70^ estimated that the photoexcited S_2_(^1^ππ*) state relaxes to the S_1_(^1^nπ*) in 201 fs, in good agreement with experiments, but the study does not extend beyond ultrafast time scales. In contrast to Borin et al. and Marian et al., who predict planarity of the molecule during S_2_/S_1_ IC, Nanbu et al. observe a twisting of the C5=N6 and N1–N2 bonds. They also identify IC to the ground state as a minor deactivation channel. On longer time scales, Marian et al. report population of the T_1_(^3^ππ*) within 125 ps in the gas phase and 30 ps in acetonitrile solutions. The increase in ISC rate in solution seems counterintuitive given that dissipative pathways can remove excess vibrational energy to the environment. On the other hand, in an isolated molecule the energy remains trapped and eases crossing of potential barriers along the deactivation pathway. According to Marian et al. a favorable solvent induced shift of the ^1^nπ* potential positions the S_1_/T_1_ crossing point toward the S_1_(^1^nπ*) minimum enabling efficient ISC. However, Borin et al. also predict near barrierless S_1_/T_1_ ISC for the isolated molecule. Once populated through either of the two pathways mentioned above, the T_1_(^3^ππ*) is long-lived due to small SOC (2.7 cm^–1^) and a 0.23 eV barrier to access the T_1_/S_0_ crossing point. The S_1_/T_1_ and T_1_/GS ISC coordinates involve a loss of planarity but Marian et al. and Borin et al. disagree whether the T_1_(^3^ππ*) minimum resembles a V-shaped (folded along N3–N6 axis) or planar structure, respectively. On nanosecond and longer time scales, dissipation of excess energy gains significance and solvent effects on the ISC rate to the ground state may become even more apparent. However, detailed evaluation of these effects requires gas-phase spectroscopic studies to isolate intrinsic photophysical behavior from environmental influences.

The purpose of the present study is to extend the earlier solution-phase time-resolved experiments on 6AU into the gas phase and verify the photophysical models and mechanistic details through a more direct comparison to theoretical predictions. To this end, we employed time-resolved photoelectron spectroscopy (TRPES) to investigate the excited state dynamics of isolated 6AU. Experiments were performed at several different excitation wavelengths across the first band of the 6AU absorption spectrum, allowing us to evaluate the effect of excess vibrational energy on the relaxation dynamics of the first bright excited state. This provides insight into the characteristic features of the excited state topographies such as barrier heights and the energies required to access crossing points along active relaxation pathways. Furthermore, we distinguish intrinsic versus environmental effects through comparison to the solution phase photodynamics.

## 2 Experimental Setup and Calculations

### 2.1 Materials

6AU was a crystalline solid purchased from Sigma-Aldrich with an assay ≥98% and used without additional purification.

### 2.2 Apparatus

A detailed description of the laser system and magnetic bottle spectrometer for the experimental setup has been reported previously.^16,71,72^ Briefly, the laser beam from the Coherent Inc. Legend Elite HE amplified laser system (central wavelength 800 nm, 1 kHz repetition rate) was utilized to generate the pump and probe beams. A tunable optical parametric amplifier (OPA), Coherent Inc., OPerA, produced the pump pulses (243–284 nm, 1.6–2.3 μJ) while the probe beam (300 nm, 4.0–4.5 μJ) was derived from the Lightconversion TOPAS-C. The selection of pump and probe wavelengths was based on the absorption spectrum of 6AU, which will be described later in the Results and Discussion section. To control the delay between the pump and probe pulses, the probe pulse was passed through an Ultrafast Systems Smart Delay Line with a 3 ns scan range. Both pulses were then focused into the ionization region of the spectrometer with the help of 50 cm convex lenses. The overlap and focusing conditions between the two beams were adjusted to optimize the signal level. The sample powder was placed inside a quartz holder, which was heated to ∼150–155 °C, and the sample vapor was coexpanded with a He carrier gas through a nozzle pinhole. The gaseous molecular beam was doubly skimmed in differential pumping stages before intersecting with the focused pump and probe beams inside the photoelectron spectrometer. Photoelectron spectra were recorded in small pump–probe delay steps near time zero and in increasing steps at longer delays. The LabView-based scan program allows the control of mechanical shutters and scan parameters for the measurement of pump-only, probe-only, and pump–probe signals. Any slow drifts in the molecular beam conditions and the laser powers were compensated by taking the average of the four bidirectional measurement sweeps. Then the one-color photoionization signals of the pump and probe beams were subtracted from the two-color ionization in order to remove the time-independent background signal. TRPES measurements of butadiene (BD) were performed for timing and energy calibrations. An instrument response function (IRF) of ∼150 fs was determined that only slightly varies with the pump wavelength. The recorded time-of-flight spectra were converted into the photoelectron kinetic energy spectra using calibration constants that were obtained in reference to cationic vibrational features of BD. The TRPES data was analyzed in two steps: initially, the integrated photoelectron signals were fit in OriginPro (OriginPro v.2019, OriginLab Corporation, Northampton, MA, USA) with sequential exponential decay equations. Three decay parameters were sufficient to adequately describe the data, but some of the data toward the onset of the absorption spectrum required an additional Gaussian function to account for a signal spike during the cross-correlation time. The extracted time constants were then utilized as an initial guess for the complete kinetic model analysis using Glotaran.^73^ Time constants and evolution-associated spectra (EAS) were obtained from the global analysis. Several effects could potentially cause the cross-correlation spike at longer wavelengths. For example, nonresonant or probe-pump signals may become more apparent as the absorption of the pump decreases. It is also possible, that the global analysis can no longer capture the shifting photoelectron band with a single time-constant as τ_1_ becomes longer than the IRF.

Gas-phase absorption spectra were measured in a home-built UV–vis spectrometer consisting of a static 35 cm long gas cell, a Hamamatsu L2D2 deuterium lamp, and an Ocean Optics HR4000 spectrometer. The sample is placed directly into the cell and heated to a similar temperature as in the TRPES experiments. Transmission spectra were recorded before any sample deposits on the windows became visible. Background spectra were obtained prior to heating the sample, but with the cell evacuated and otherwise identical experimental conditions. The gas-phase absorption spectrum is then derived by subtracting the background from the transmission spectra.

### 2.3 Calculations

To interpret the experimental spectra, knowledge of vertical excitation and ionization energies is essential. Single point energy calculations were performed at the XMS-CASPT2­(16,11)/ANO-R2 level of theory in OpenMOLCAS v24.06,^74^ replicating the optimized geometries for critical points and minima of the relevant neutral states from Ref 65. The active space orbitals used in these calculations are visualized in Figure S1. The XMS-CASPT2 calculations were carried out using the corresponding CASSCF wave functions and employing an IPEA shift of 0.25 au and an imaginary shift of 0.1 au These shifts were introduced to avoid the contribution of intruder states which can otherwise reduce the accuracy of the results. Vertical excitation and ionization energies and the orbital contributions to the electronic state character are collated in Table S1 in the Supporting Information. Vertical excitation energies for the 10 lowest singlet excited states are evaluated to gain insight into their electronic character and contributions to the steady-state absorption spectrum. Ionization potentials (IPs) along the deactivation path are evaluated with respect to the energy of the neutral ground state minimum. For each singlet and triplet state minimum, the IPs are calculated as the sum of the adiabatic excitation energy and vertical ionization energy from that minimum. The extent of overlap between the electron wave functions of the neutral state and the resulting cationic state is described as a Dyson orbital. The associated Dyson intensities, i.e., Dyson norms (see Table S2), quantify the ionization probabilities for each state and are used to identify the most probable ionization channel. Spectral shifts in the photoelectron spectra or EAS are evaluated by considering the vibrational energy gain during electronic relaxation. This is estimated as the difference in vertical energy of the photoexcited state and adiabatic energies of the excited state minima along the deactivation path. This vibrational excitation is assumed to be transferred to the cation upon ionization and therefore added to the IP. The ionization energy to the vibrationally excited cationic state is referred to as photoelectron binding energy (eBE) throughout the manuscript. These estimates are summarized in Table S3 in the Supporting Information.

## 3 Results and Discussion

### 3.1 Absorption Spectrum and Electronic States

Figure 2 depicts the gas-phase UV/vis absorption spectrum of 6AU which exhibits two main absorption bands centered near 250 nm and below 200 nm. Overall, its shape resembles the gas-phase spectrum of uracil, except for a 0.1 eV (5 nm) redshift of the first absorption band. The maximum of the second band is blueshifted beyond the measured wavelength range. For the present study the primary interest is the first absorption band and insight into the underlying excited state transitions is gained from the XMS-CASPT2 (16,11)/ANO-R2 calculations. The excitation energies, nature of orbital transitions, and oscillator strengths of the lowest singlet and triplet states of uracil and 6AU are summarized in Table 1. Additional calculations for higher states are provided in the Table S1. In the case of uracil, the first absorption band with a maximum at 5.04 eV is attributed to a bright ^1^ππ* transition. In 6AU, the calculated energy of 5.10 eV for the first bright ^1^ππ* state, S_2_, with sizable oscillator strength, matches the maximum of the first absorption band at 4.95 eV reasonably well. Therefore, the calculations confirm that, similar to uracil, the first absorption band is associated with the S_2_(^1^ππ*) excited state. This transition is derived from the promotion of an electron from the π orbital (HOMO), which is mostly localized on the double bond in the ring, to the π* orbital (LUMO) as shown in Figure 2. The lowest singlet S_1_(^1^nπ*) excited state has a small oscillator strength rendering it optically mostly dark and is located vertically 0.35 eV below the S_2_(^1^ππ*) excited state. This state primarily originates from the HOMO – 1 orbital with contributions from the lone pair electrons on the N6 atom. Compared to uracil, the ordering of the lowest ^1^nπ* and ^1^ππ* excited states remains the same but additional delocalization over the N atom substituent causes an overall energetic lowering of the S_1_(^1^nπ*). The small energy gap between the S_1_(^1^nπ*) and the T_1_(^3^ππ*) states in the S_1_/T_1_ crossing region, which is located close to the S_1_(^1^nπ*) minimum, combined with favorable SOC accounts for the distinctly different relaxation pathways and enhanced ISC in 6AU compared to the U.^65,66^

**Figure 2:**
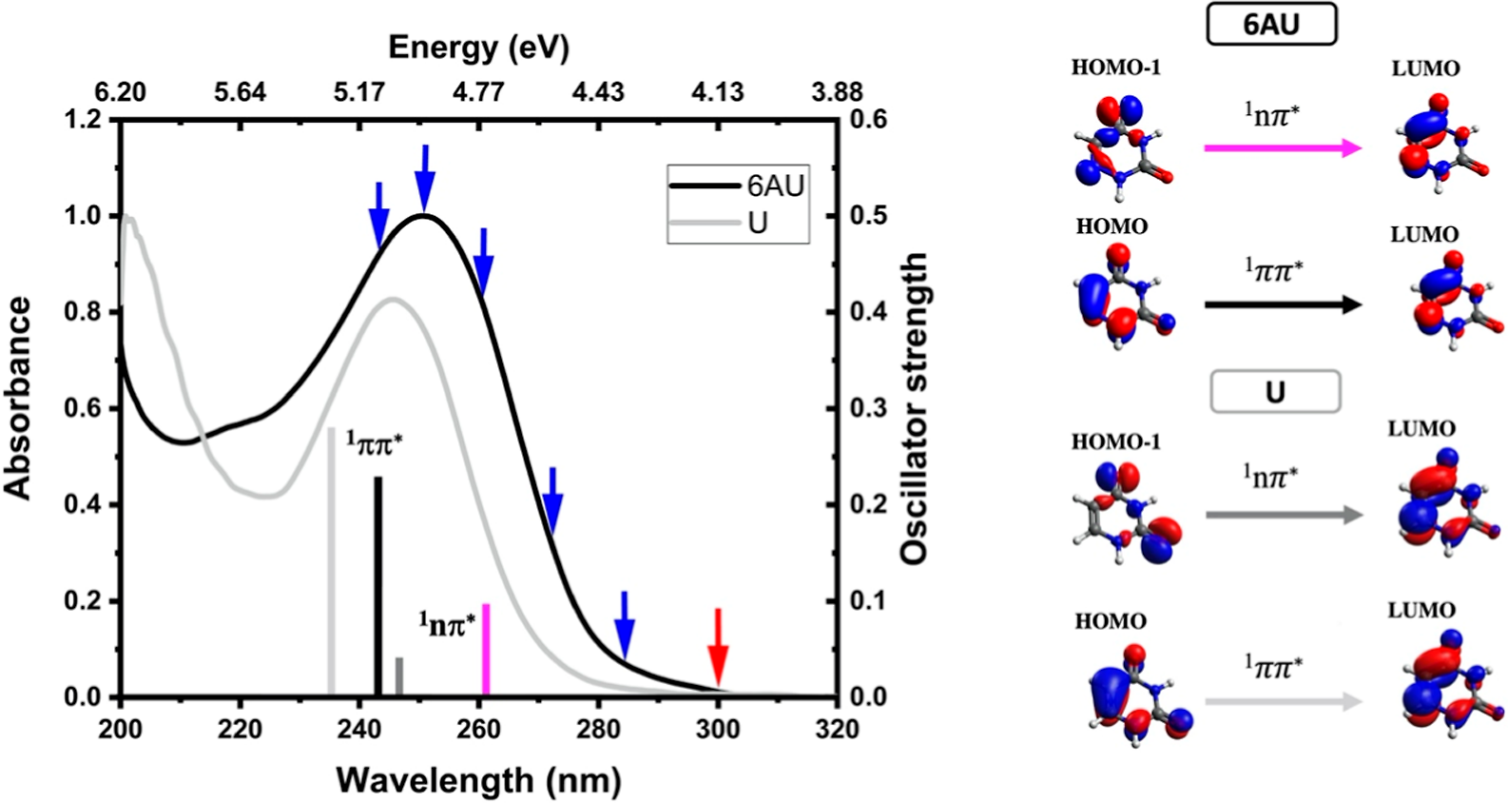
Experimental UV–vis absorption spectrum of 6AU in the gas phase where blue and red arrows indicate the pump and probe wavelengths. The vertical black and magenta lines represent the calculated vertical excitation energies for the ^1^ππ* and ^1^nπ* singlet states, respectively, with the lengths indicating the oscillator strengths. For the ^1^ππ* state the length directly corresponds to the specified values in Table 1, while for the ^1^nπ* state a multiplication factor of 100 was applied. The uracil absorption spectrum is shown as a light gray curve for comparison. The corresponding ^1^ππ* and ^1^nπ* states are marked by light and dark gray solid lines with the same length scaling as 6AU. The orbital transitions associated with the S_1_(^1^nπ*) and S_2_(^1^ππ*) states are derived from the n → π* and π → π* transitions shown on the right. The orientations of the 6AU and U molecular structures are consistent with those in Figure 1.

**Table 1:** Vertical Excitation Energies and Oscillator Strengths for the Calculated Singlet and Triplet States of 6AU and U^a^

states	orbital nature	energy (eV)		oscillator strength	
		6AU	U	6AU	U
S_1_	nπ*	4.75	5.04	0.00102	0.00039
S_2_	ππ*	5.10	5.27	0.23045	0.28411
S_3_	nπ*	5.59	6.44	0.00468	0.00021
S_4_	ππ*	5.92	6.33	0.08318	0.05750
T_1_	ππ*	3.82	4.04		
T_2_	nπ*	4.30	4.92		
T_3_	ππ*	5.26	5.51		
T_4_	nπ*	4.37	6.34		

a Values for *U*, calculated at the same level of theory, are provided for comparison.

### 3.2 TRPES Measurements and Excited State Dynamics

The colormaps of the TRPES signals and results of their analysis are shown in Figure 3 in ascending order of excitation wavelength from the top to the bottom row, i.e. from bottom to top the 6AU molecules are photoexcited with an increasing photon energy. In the first column, colormaps of the photoelectron signals are plotted as eBE (eV) versus pump–probe delay time (ps). During the temporal overlap of the pump and probe pulses, the system is excited vertically to the Franck–Condon (FC) region and ionized immediately. These instantaneous TRPES spectra can be compared to a measured He­(I) photoelectron spectrum for insight into potential ionization channels. The first four peaks in the He­(I) spectrum of 6AU are located in the energy range 10.18 to 11.14 eV and attributed to ionization from the two in-plane oxygen lone pair orbitals (n^–1^-holes) and two π^–1^-hole molecular orbitals on the enaminic and dicarbonyl moieties.^75^ A higher energy ionization peak at 12.68 eV corresponds to the nitrogen lone pair orbital (n^–1^-hole). For the TRPES, this implies that vertical ionization of the intermediate excited states requires a two-photon ionization process and a (1 + 2′) excitation-ionization scheme is assumed in the conversion from measured electron kinetic energies to eBE. As can be seen in the colormaps in Figure 3 this assumption places the photoelectron signal in the correct energy range.

**Figure 3:**
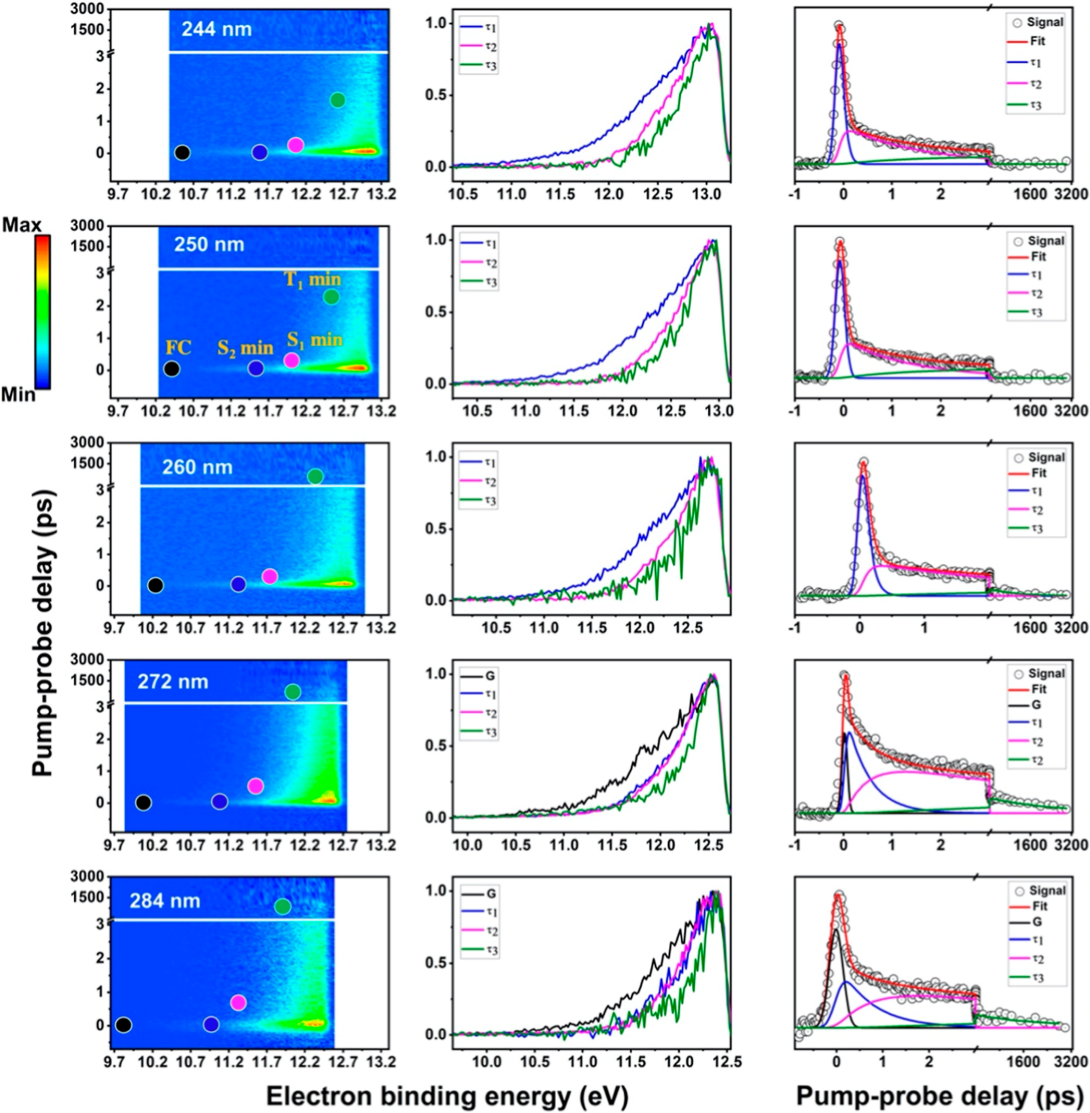
TRPES and fit results from global analysis with a sequential exponential decay model. The first column displays the colormaps of the experimental TRPES and the superimposed dots represent the calculated eBE for ionization from the FC region and the excited state minima as labeled in the figure. Each row contains the fit results for a given wavelength. In the EASs (column 2) the black/blue curve represents the depopulation dynamics of the photoexcited state S_2_(^1^ππ*), the magenta curve shows the rise and decay of the S_1_(^1^nπ*) population, and the green curve captures the population changes in the lowest triplet T_1_(^3^ππ*). Columns 3 contains the energy-integrated signal, total fit and the contributions from individual components.

Some general wavelength-dependent trends can be observed. For example, shorter excitation wavelengths cause the entire TRPES spectrum to systematically shift toward higher eBE. Based on the UV–vis absorption spectrum, all pump wavelengths photoexcite 6AU to the lowest bright electronic state, S_2_(^1^ππ*). Direct photoexcitation of the S_1_(^1^nπ*) can be excluded based on its low oscillator strength. Although in the solution phase Kobayashi et al.^67^ have reported direct excitation of the S_1_(^1^nπ*) at 308 nm, the TRPES show no sign of probe-pump signals from single-photon excitation with the 300 nm probe pulse. Hence, all different pump wavelengths, including 272 and 284 nm, are assumed to photoexcite the S_2_(^1^ππ*) but with different amounts of vibrational excitation. The overall shift in the TRPES is consistent with the excess excitation energy in the S_2_(^1^ππ*) and attributed to a transfer of excited state vibrational energy to the cation during photoionization. This simplistic model of a Δ*v* = 0 propensity is therefore applied in the subsequent analysis and interpretation. Furthermore, as the total (pump plus probe) photon energy increases, the cutoff of the photoelectron spectra extends toward higher eBE. The general shape of the TRPES spectrum, which is indicative of the relaxation pathway, is preserved, suggesting that no significant change in the deactivation mechanism occurs as the excess energy increases.

Further insight into the mechanism can only be gained through thorough analysis and interpretation of the time-dependent shifts in the TRPES which is presented below for the 250 nm data and then extrapolated to other wavelengths. The initial analysis of the energy-integrated photoelectron signals showed a good fit of the data with a sequential exponential decay function with three components, whereas inclusion of an additional Gaussian function was only necessary for 272 and 284 nm excitation (which is discussed further below). With these considerations and using the extracted timeconstants as initial guesses, global lifetime analysis was conducted for each TRPES data set. The results are presented in Figures S7 and S8 of the Supporting Information where each colormap plot represents the time-evolution of the photoelectron band associated with a fit component (i.e., an ionization channel). The time- and energy-integrated plots in Figure 3 columns 2 and 3 are all derived from these components. Column 3 shows the integrated timetrace of the photoelectron signal, along with the total fit and the individual components. The extracted decay timeconstants are collated in Table 2 and the evolution-associated spectra (EAS) are plotted in column 2. The adequacy and quality of the fits is confirmed by random and negligible residuals that remain after the analysis and singular value decomposition (see Supporting Information for further discussion). This fitting model also supports a three-step relaxation route as proposed by the theoretical calculations.^65,66^

**Table 2:** Decay Time Constants Extracted From the TRPES Data through Global Analysis with a Three Exponential, Sequential Decay Model^a^

wavelength (nm)	τ_1_ (fs)	τ_2_ (ps)	τ_3_ (ns)
244	<100	1.26	0.046
250	<100	2.22	0.171
260	101	3.24	1.04
272	469	5.28	2.67
284	729	6.07	2.79

a An additional Gaussian function was included in fits of the 272 and 284 nm TRPES only. The error is estimated as ± 15% based on the analysis of several data sets and represents the reproducibility of the time constants.

Generally, the decay times represent the population dynamics of a specific state and the EASs are characteristic of the states’ orbital nature. The latter require further interpretation based on ab initio calculations of the excitation and ionization energies, orbital contributions, and Dyson intensities for distinct ionization channels from the electronically excited states. This information is captured in Table S1 of the Supporting Information and Figure 4 demonstrates how it is applied in the interpretation of the TRPES. The dots with a white border superimposed on the colormap indicate the calculated eBE where a photoelectron band associated with ionization from a specific state is expected to appear. For example, 250 nm (4.96 eV) excitation is close to the maximum of the UV–vis absorption band. The calculated eBE for ionization from the FC region is 10.43 eV and indicated by a black dot in Figure 4b. This coincides with the onset of the photoelectron band. Evolution from the FC region to the S_2_(^1^ππ*) minimum, which is located at 4.66 eV above the ground state, is associated with a vibrational energy gain of about 0.44 eV and an increase in ionization potential to 11.10 eV for the D_0_(π^–1^) cationic state. The estimated eBE for ionization from the S_2_(^1^ππ*) minimum is 11.54 eV. This causes a significant shift of the photoelectron band toward higher eBE at early pump–probe delays. In Figure 4a this is clearly visible as a signal that starts around 10.43 eV and extends beyond 13 eV. Global analysis extracts an ultrafast component as shown in Figure 4b which is associated with the initial evolution out of the FC region and the population decay dynamics of the S_2_(^1^ππ*) state. The initial relaxation occurs rapidly and well within the IRF of the experiment resulting in weak signals. The ionization potentials for S_1_(^1^nπ*) and the lowest triplet state, T_1_(^3^ππ*), are calculated as 10.90 eV for the D_0_(n^–1^) hole and 10.64 eV for D_0_(π^–1^) hole, respectively. With these ionization potentials and an estimated vibrational excitation of 1.00 and 1.88 eV, the photoelectron bands are expected at eBEs of 11.90 eV (magenta dot) and 12.52 eV (green dot), respectively. These predictions are in good agreement with the experimental observation, i.e. the components plotted in Figures 4c and 4d fall within the eBE range indicated by the dots for ionization from the S_1_(^1^nπ*) and T_1_(^3^ππ*) states. Based on this analysis a model for the relaxation mechanism can be derived: Excitation with the applied 250 nm pump pulse launches the molecules into the first bright S_2_(^1^ππ*) state which decays with a timeconstant of less than 100 fs to an intermediate state, S_1_(^1^nπ*). The latter has a lifetime of 2.22 ps and is depopulated by ISC to a long-lived (0.171 ns) triplet state, T_1_(^3^ππ*).

**Figure 4:**
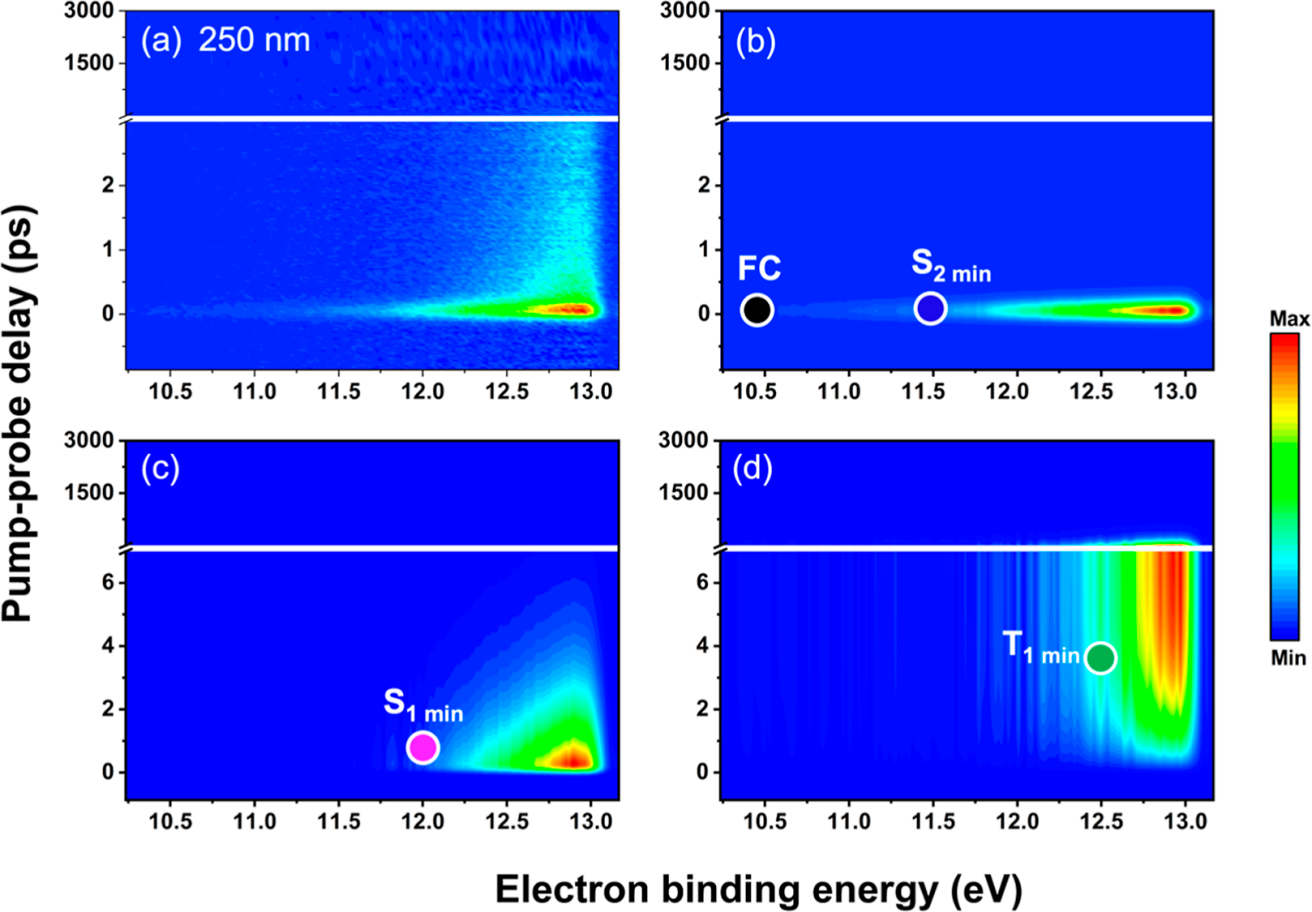
TRPES (a) recorded at 250 nm excitation and three components (b–d) extracted from global analysis are represented in terms of eBE versus delay time. The dots mark the eBE range where the photoelectron bands for ionization from the S_2_–FC—black, S_2_(^1^ππ*)—blue, S_1_(^1^nπ*)—magenta, and T_1_(^3^ππ*)—green are expected.

A similar analysis and interpretation is performed for all other excitation wavelengths. Consistent with the results for 250 nm excitation, the population dynamics are best described by a sequential decay model with three exponentials. The extracted timeconstants are summarized in Table 2. A clear wavelength-dependent trend is observed such that the decay times of the excited S_2_(^1^ππ*), S_1_(^1^nπ*), and T_1_(^3^ππ*) states increase with an increase in the excitation wavelength, i.e. the lifetimes of the excited states become longer as vibrational excitation is reduced.

The normalized EAS (Figure 3 column 2) and the component plots (Figures S7 and S8) maintain a similar spectral shape independent of the excitation wavelength. The systematic trend in the decay times combined with a consistent EAS indicates that the 250 nm model is transferable to other wavelengths and the relaxation mechanism remains the same. However, excess vibrational energy eases barrier crossing and access to crossing points with lower electronic states. Consequently, the nonradiative decay rates are expected to increase toward shorter excitation wavelengths which is also observed.

The inclusion of an additional Gaussian fit component for the longer wavelengths TRPES requires further elaboration. Figures S7 and S8 in the SI provide insight as to the origin of the underlying processes. At excitation wavelengths of 260 nm and shorter, relaxation out of the FC region and depopulation of the S_2_(^1^ππ*) occur on time scales close to the experimental IRF and can therefore be captured by a single timeconstant, τ_1_. However, as the vibrational excess energy is reduced the ultrafast dynamics occur on time scales of several hundred femtoseconds. At 272 and 284 nm excitation, two separate timeconstants, G and τ_1_, are needed to describe the initial motion toward the S_2_(^1^ππ*) minimum and the subsequent depopulation dynamics of this state, respectively. This interpretation is supported by the EAS which are plotted as component colormaps in Figure S8. The spectrum associated with G extends over a broad eBE range starting from FC (black dot), whereas the τ_1_ spectrum is narrower and falls into the estimated eBE range of the S_2 min_ (blue dot). For shorter excitation wavelengths (Figure S7), the colormaps of the τ_1_ EAS extend to the eBE associated with the FC region. Although the fitting of the initial dynamics differ, the photophysical deactivation mechanism remains the same for all excitation wavelengths. Alternative explanations for G, such as direct photoexcitation of the S_1_(^1^nπ*) can be excluded based on the absence of any probe-pump signals in the TRPES which would correspond to a 300 nm excitation with the probe.

### 3.3 Comparison to Theory

The TRPES results are used to evaluate the two relaxation routes proposed by theory:^65,66^ S_2_(^1^ππ*) → S_1_(^1^nπ*) → T_1_(^3^ππ*) versus S_2_(^1^ππ*) → T_2_(^3^nπ*) → T_1_(^3^ππ*). Initial photoexcitation at all pump wavelengths is to the bright S_2_(^1^ππ*) state. According to Borin et al.^65^ a 0.55 eV high barrier separates the S_2_(^1^ππ*) minimum from the CI with the ground state. At the shortest excitation wavelengths, the photon energy becomes sufficient to surpass this barrier. However, this would require that all vibrational excess energy flows into the deactivation coordinate toward the transition state with a C5=N6 twist and steep H5 out-of-plane bend. On the basis of the observed ultrafast τ_1_ timeconstants direct S_2_ → S_0_ IC is therefore considered unlikely. The systematic decrease of τ_1_ is more consistent with the presence of a small barrier. The S_2_/S_1_ and S_2_/T_2_ crossing points are located close to the S_2_(^1^ππ*) minimum and provide a more accessible avenue for deactivation on ultrafast time scales. The proposed S_2_ → T_2_ ISC has to compete with S_2_ → S_1_ IC that does not require a spin flip. Given the relatively low SOC of about 16–17 cm^–1^,^65,66^ and non-negligible singlet–triplet gap of 0.16 eV, S_2_ → T_2_ ISC will play an insignificant role. Nanbu and Iwasa^70^ used on the fly molecular dynamics simulations that identify ultrafast IC as the main pathway and provide a timeconstant of 201 fs for relaxation from the FC region to S_1_(^1^nπ*) in good agreement with the observed τ_1_ from the 250 nm TRPES. Borin et al. and Nanbu et al. however disagree on the relaxation coordinates, i.e. planar versus twisting around the C5=N6 and N1–N2 bonds, respectively. The observed wavelength-dependent trend in τ_1_ suggests that a small potential barrier exists along the IC coordinate and such a barrier has also been reported by Borin et al. A barrier height of 0.2 eV with respect to the S_2_(^1^ππ*) minimum is calculated along the minimum energy path. Furthermore, radiative depopulation of the S_2_(^1^ππ*) has been excluded by Marian et al., who estimate a fluorescence lifetime of 160 ns, which is too long to effectively compete with ultrafast IC. On the basis of the experimental observation and agreement with theory, the first step in the relaxation process is attributed to evolution out of the FC region followed by IC from the S_2_(^1^ππ*) minimum to the S_1_(^1^nπ*).

The second step involves the depopulation dynamics of the S_1_(^1^nπ*) and three different processes are considered: IC to the ground state, ISC to T_1_(^3^ππ*), or radiative decay. The observed τ_2_ timeconstants are on the order of a few picoseconds and show a trend indicative of a small barrier along the relaxation coordinate. These observations are incompatible with a ∼ 1 eV barrier from the S_1_(^1^nπ*) minimum to the CI with the ground state predicted by Borin et al. While radiative decay from the S_1_(^1^nπ*) has not been characterized computationally, ISC is expected to be dominant. The S_1_/T_1_ crossing point can be reached by surmounting a small to negligible barrier along in-plane distortions surrounding the C4 position of the ring and out-of-plane bending of the N3–H bond.^65,66^ Furthermore, ISC follows El Sayed’s rule and is efficient due to high SOC (64.7 cm^–1^). This second step is therefore associated with S_1_(^1^nπ*) → T_1_(^3^ππ*) ISC. It should however be noted that the observed timeconstants are significantly shorter than the 125 ps estimated by Marian et al.

The third timeconstant, τ_3_, falls within a long picosecond to nanosecond time frame and shows a significant wavelength dependence. Both Marian et al. and Borin et al. predict negligible SOC at the crossing point with the ground state. Furthermore, a barrier is located along the relaxation path, but there is disagreement as to the barrier height, i.e. 0.2 eV versus ∼0.5 eV according to Borin et al. and Marian et al., respectively. The latter, however, is based on a linear extrapolation of the relaxation coordinate which is known to overestimate barriers compared to minimum energy path calculations. Additionally, the T_1_/S_0_ crossing point geometry is characterized by a substantial out-of-plane distortion at the C5–H5.^65^

Based on the discussion above S_2_(^1^ππ*) → S_1_(^1^nπ*) → T_1_(^3^ππ*) is considered the dominant pathway. Unfortunately, the EAS do not provide any additional evidence in support of this pathway and exclusion of the other because the photoelectron bands for ionization from S_1_(^1^nπ*) and T_2_(^3^nπ*) are expected to fall within the same eBE range (see Table S3). They are therefore indistinguishable.

### 3.4 Comparison to Solution-Phase Dynamics of 6AU

Similar to the gas-phase dynamics, transient absorption studies on 6AU in acetonitrile solution observe a three-step sequential exponential decay. For 264 nm photoexcitation, Liu et al.^76^ report timeconstants of <0.3 ps, 5.2 ps, and >1000 ps that are assigned to S_2_(^1^ππ*) → S_1_(^1^nπ*) IC, S_1_(^1^nπ*) → T_1_(^3^ππ*) ISC, and prolonged trapping in T_1_(^3^ππ*), respectively. The triplet state lifetime was measured by Kobayashi et al.^77^ using picosecond transient absorption spectroscopy and τ_3_ was determined to be ∼190 ns long. These solution phase timeconstants are consistent with the values from TRPES, except for a much longer-lived triplet state. ISC back to the ground state occurs significantly faster in vacuum than in the solution phase. This can be rationalized by the dissipation of excess energy to the solvent environment, whereas in isolated 6AU a vibrationally hot triplet state maintains an internal energy that is sufficient to overcome the barrier to the T_1_/S_0_ crossing point and samples the C5–H5 out-of-plane deformation of the crossing point geometry more frequently. In summary, it can be concluded that the general deactivation mechanism is preserved in the solution environment. As shown by Liu et al. this model can be extended to different solvents and there is a polarity dependence of the deactivation times. In solvents with high polarity S_1_(^1^nπ*) → T_1_(^3^ππ*) ISC is faster but still falls within the few ps range. However, some aspects of the ISC dynamics and mechanisms remain contradictory. For example, Marian et al. predicted S_1_(^1^nπ*) → T_1_(^3^ππ*) ISC to be significantly faster in the solution phase than in vacuum (30 ps in acetonitrile versus 125 ps in vacuum), however the experimentally observed timeconstants are almost identical. Radiative decay, as an alternative route to ground state repopulation, has been found to play only a negligible role.^77^

### 3.5 Comparison to Uracil Photodynamics

For photoexcitation of uracil at 260 nm, which is on the rising edge of the absorption spectrum, Yu et al.^32^ reported timeconstants of τ_1_ = 170 fs, τ_2_ = 2.35 ps, and τ_3_ > 1 ns. Over the 500 ps observation window the τ_3_ signal shows no decay and the final state can be considered long-lived. Similar to 6AU, these timeconstants represent the depopulation dynamics of the S_2_(^1^ππ*), S_1_(^1^nπ*), and lowest triplet state, T_1_(^3^ππ*). The 6AU TRPES with 272 nm excitation introduces a similar amount of vibrational excess energy into the bright S_2_(^1^ππ*) and is therefore used for comparison here. The 6AU timeconstants of τ_1_ = 469 fs, τ_2_ = 5.28 ps, and τ_3_ = 2.67 ns show slightly slower decays of the S_2_(^1^ππ*) and S_1_(^1^nπ*) states but the T_1_ → S_0_ deactivation is faster. This observation can be explained by theoretical models and distinctly different deactivation mechanisms. The optimized geometry of both uracil and 6AU has a planar structure at its ground state minima. The N-substitution does not have a significant effect on the C=O bond lengths, but it perturbs the electron delocalization and resonance structure of the ring bonds and, overall, lowers the vertical excited state energies of the S_2_(^1^ππ*) and more notably of the S_1_(^1^nπ*) state (Table 1, Figure 2). In uracil depopulation of the initially excited S_2_(^1^ππ*) state is facilitated by several CIs, either along planar or ethylenic coordinates, that lead directly back to the ground state or the lower-lying S_1_(^1^nπ*) state. The rigidity of the C5=N6 bond in 6AU, compared to the C5=C6 bond in uracil, quenches the relaxation routes through the ethylenic CIs. Furthermore, the lone pair of the substituent N atom contributes to the HOMO – 1, which takes part in the n → π* transition in 6AU, and induces state specific electronic shifts to minima and crossings. Similar but more subtle than in thiobases, the lowering in the vicinity of the singlet state minima is more pronounced than at the CIs which introduces high barriers that block direct access to the ground state. This efficiently funnels all excited state population into the S_1_(^1^nπ*) through a CI that is located close to the S_2_(^1^ππ*) minimum. The restrictions on ethylenic pathways but nearly barrierless IC path to S_1_(^1^nπ*) explains the observation of slower, but still ultrafast, τ_1_ dynamics in 6AU. In the case of uracil, barriers to access CIs for S_2_(^1^ππ*) depopulation are small to negligible, but strongly depending on the level of theory which prevents a more quantitative comparison.^62,78,79^ The S_1_(^1^nπ*) of uracil can undergo a branching decay with IC to the ground state via an ethylenic CI and ISC to T_1_(^3^ππ*). Only the latter pathway is available in 6AU. The computed SOC for S_1_(^1^nπ*) → T_1_(^3^ππ*) ISC is 16 cm^–1^ and 64.7 cm^–1^ for uracil and 6AU, respectively. Despite the lower SOC in uracil, the τ_2_ timeconstant associated with S_1_(^1^nπ*) depopulation is shorter in uracil because it represents a weighted average of the competing decay paths with a dominant contribution from IC to the ground state. In 6AU, in contrast, the τ_2_ timeconstant describes ISC only but is more efficient due to a negligible barrier and higher SOC at the S_1_/T_1_ crossing. In both molecules, the T_1_(^3^ππ*) population gets trapped for nanoseconds due to low SOC (1–2 cm^–1^) at the T_1_/S_0_ crossing, but the barrier in 6AU is lower (0.2 eV in 6AU^65^ versus 0.4 eV in Uracil^32^) and hence a shorter triplet lifetime, τ_3_, is expected. This comparison highlights the significantly different deactivation mechanisms in 6AU and U despite their decay time scales being of similar order of magnitude. Furthermore, single atom substitution of the C6 in the ring with N effectively enhances ISC. In condensed-phase experiments on 6AU and U under the same conditions, it was shown that the quantum yield of ISC for 6AU is near unity, whereas it is only 0.21 for uracil.^67,80^

## 4 Conclusions

The excited state dynamics of 6AU have been investigated using TRPES with pump wavelengths between 244 and 284 nm and a two-photon probe of 300 nm. The investigation reveals the existence of an efficient nonradiative decay pathway, although direct IC from the singlet excited states to the ground state as in uracil is prevented. These CIs are of ethylenic nature and N-substitution at the C6 position hinders access due to electronic (increase in barrier to CIs) and structural (increase in N6=C5 bond rigidity) aspects.^65,67^ Instead, in 6AU ISC into the triplet manifold becomes efficient. The relaxation mechanism in 6AU is summarized in Figure 5. Following initial excitation to the FC region of the S_2_(^1^ππ*) state, 6AU quickly relaxes to the S_2_ minimum and a CI with the S_1_(^1^nπ*) can be accessed by surmounting a ∼0.2 eV barrier.^65^ Either an out-of-plane twisting motion at the C5=N6 and N1–N2 bonds^70^ or planar modes^65^ lead to IC to the lower state on time scales of hundreds of femtoseconds. A crossing point to the T_1_(^3^ππ*) with SOC of 64.7 cm^–1^ is located close to the S_1_(^1^nπ*) minimum. ISC occurs within a few picoseconds and involves in-plane distortions surrounding the C4 position of the ring and out-of-plane bending of the N3–H bond.^65,66^ Population on the T_1_(^3^ππ*) triplet state stays trapped for picosecond to nanoseconds times. Although the crossing can be reached by surpassing a small (0.23 eV) barrier, the low SOC (1–3 cm^–1^) and heavily distorted crossing geometry (out-of-plane twisted C5=N6 bond with strong C5 pyramidalization) make ISC inefficient.^65^

**Figure 5:**
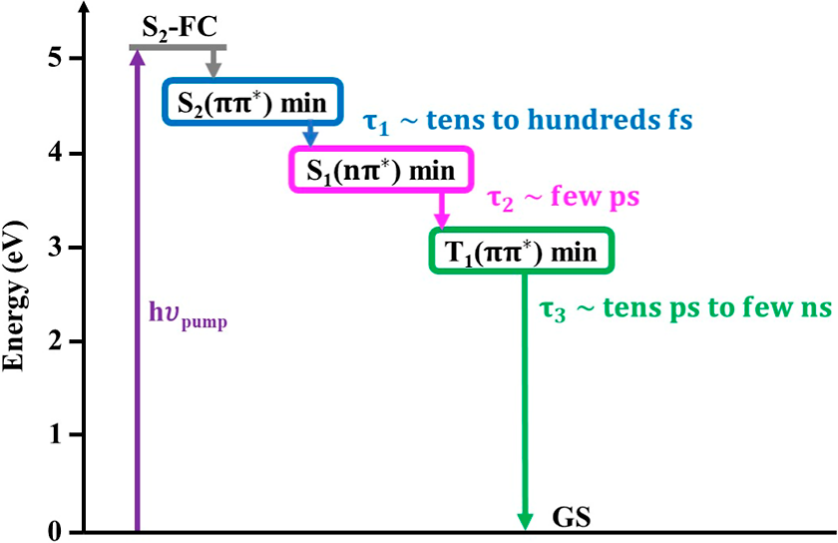
Scheme of the dominant decay pathway of 6AU following photoexcitation to its lowest bright S_2_(^1^ππ*) state. Time scales of the internal conversion and intersystem crossing dynamics are provided.

In summary, this study highlights that aza-substitution at the C6 position of uracil limits internal conversion through ethylenic CIs with the ground state and instead funnels population along an ISC pathway. While there are similarities to the photodynamics of thiouracils, the mechanistic details are significantly different. Sulfur substitution of the exocyclic oxygen also enhances ISC to a near unity quantum yield. Furthermore, the S_1_(^1^nπ*) state serves as a doorway into the triplet manifold but the relaxation coordinate involves an out-of-plane pyramidalization at the C2 position of the ring.^31^

## Supplementary Material



## Data Availability

The manuscript and Supporting Information provide the details of the calculations and fitting procedure applied to the experimental data. The raw experimental data are in a custom format and are available from the authors upon request.

## Notes

Computational details (active space orbital, energies, geometries) and further information on the data analysis and interpretation (justifications for fitting model, additional plots of the fit components obtained from global lifetime analysis, and assignments of photoelectron bands) (PDF)
