# Supplementary material for: Electronic Relaxation Dynamics of 6‑Azauracil: The Effect of Ring Substitution on Intersystem Crossing
Source: J Phys Chem A. 2025 Sep 25;129(40):9279–90. doi: 10.1021/acs.jpca.5c04895 (PMC12516718; doi:10.1021/acs.jpca.5c04895)
Supplement: Supplementary file 1 [file jp5c04895_si_001.pdf]

Supporting Information

Electronic Relaxation Dynamics of 6-Azauracil: The Effect of Ring Substitution on Intersystem Crossing

Moti Raj Chudali, Susanne Ullrich*
Department of Physics and Astronomy, University of Georgia, Athens, GA 30602, USA

*Corresponding author email: ullrich@uga.edu

ORCID: MC (0009-0005-2480-5836); SU (0000-0002-1828-2777)

Contents

1: Active space orbitals S2
2: Vertical excitation energies and orbital characters S3
3: Excitation and ionization energies along deactivation path S4
4: Cartesian coordinates S6
5: Justification of decay model S7
6: References S13

1. Active space orbitals

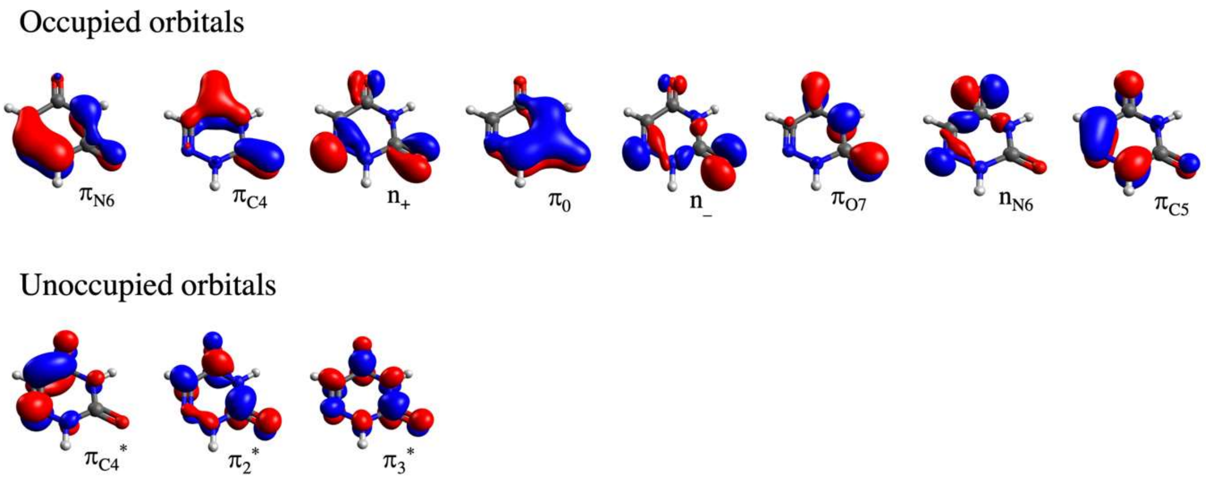

Fig. S1: CAS(16,11) singlet molecular orbitals calculated at the S_0 minimum geometry. The upper row displays the occupied orbitals while the lower row represents the unoccupied orbitals. The labels are descriptive of the orbital configurations and are meant for the purpose of further discussions.

2. Vertical excitation energies and orbital characters

Table S1: Vertical excitation energies, oscillator strengths, and orbital characters of singlet states and selected doublet and triplet states computed at the S_0 minimum geometry using XMS-CASPT2(16,11)/ANO-R2 level of theory.

	**XMS-CASPT2(16,11)/ANO-R2**				
**States**	**Vertical Excitation Energy (eV)**	**Oscillator Strength**	**Orbital characters**		
S_0	0.00	—	87%	closed shell	
S_1	4.75	1.02141086E-03	77%	$n_{N6}\rightarrow\pi_{C4}^*$	$n\pi^*$
S_2	5.10	2.30453388E-01	65%	$\pi_{C5}\rightarrow\pi_{C4}^*$	$\pi\pi^*$
S_3	5.59	4.67673386E-03	71%	$n_{-}\rightarrow\pi_{C4}^*$	$n\pi^*$
S_4	5.92	8.31852476E-02	65%	$\pi_{O7}\rightarrow\pi_{C4}^*$	$\pi\pi^*$
S_5	6.72	2.69136690E-03	36%<br>16%<br>10%	$n_{-}\rightarrow\pi_2^*$<br>$n_{+}\rightarrow\pi_{C4}^*$<br>$n_{+}\rightarrow\pi_2^*$	$n\pi^*$<br>$n\pi^*$<br>$n\pi^*$
S_6	7.17	5.48983233E-03	16%<br>35%	$n_{-}\rightarrow\pi_2^*$<br>$n_{+}\rightarrow\pi_{C4}^*$	$n\pi^*$<br>$n\pi^*$
S_7	7.83	1.86619078E-03	22%<br>35%	$\pi_{C5},n_{N6}\rightarrow\pi_{C4}^*$<br>$n_{N6}\rightarrow\pi_2^*$	$n\pi^*$<br>$n\pi^*$
S_8	8.14	2.86434079E-02	12%<br>30%<br>21%	$\pi_{C5},\pi_{C5}\rightarrow\pi_{C4}^*$<br>$\pi_{C5}\rightarrow\pi_2^*$<br>$\pi_{C4}\rightarrow\pi_2^*$	$\pi\pi^*$<br>$\pi\pi^*$<br>$\pi\pi^*$
S_9	8.83	3.57955788E-04	38%<br>17%<br>17%	$n_{N6},n_{N6}\rightarrow\pi_{C4}^*$<br>$n_{-},n_{N6}\rightarrow\pi_{C4}^*$<br>$\pi_{C4},n_{N6}\rightarrow\pi_{C4}^*$	$n\pi^*$<br>$n\pi^*$<br>$n\pi^*$
D_0	10.43	—	78%	π_{C5}	π^{-1}
D_1	10.56	—	66%<br>11%	n_{N6}<br>n_{-}	n^{-1}<br>n^{-1}
D_2	11.06	—	58%<br>11%	n_{-}<br>n_{+}	n^{-1}<br>n^{-1}
D_3	11.26	—	67%	π_{O7}	π^{-1}
D_4	12.77	—	57%	n_{+}	n^{-1}
T_1	3.82	—	76%	$\pi_{C5}\rightarrow\pi_{C4}^*$	$\pi\pi^*$
T_2	4.30	—	32%<br>32%<br>16%	$n_{N6}\rightarrow\pi_{C4}^*$<br>$n_{-}\rightarrow\pi_{C4}^*$<br>$n_{+}\rightarrow\pi_{C4}^*$	$n\pi^*$<br>$n\pi^*$<br>$n\pi^*$
T_3	5.27	—	64%	$\pi_{O7}\rightarrow\pi_{C4}^*$	$\pi\pi^*$
T_4	5.33	—	38%<br>27%	$n_{-}\rightarrow\pi_{C4}^*$<br>$n_{+}\rightarrow\pi_{C4}^*$	$n\pi^*$<br>$n\pi^*$

3. Excitation and ionization energies

Table S2: Ionization energies, orbital characters, and Dyson intensities for ionization from different excited state geometries. Ionization energies are calculated at the same level of theory mentioned above. The ionization energies are in units of eV and provided relative to the energy of the S_0 minimum. Ionization channels with high Dyson intensities, which are expected to dominate the photoelectron spectra, are indicated in bold.

Geometry	D_0	D_1	D_2	D_3
**S_2 (ππ*) FC**	**10.43 (π^{-1})**	10.56 (n^{-1})	11.06 (n^{-1})	11.26 (π^{-1})
$S_2 \rightarrow D_x$ intensity	**4.43E-01**	1.37E-02	3.02E-02	2.17E-02
**S_1 min (nπ*)**	**10.90 (n^{-1})**	11.07 (π^{-1})	12.08 (π^{-1})	**12.42 (n^{-1})**
$S_1 \rightarrow D_x$ intensity	**4.19E-01**	1.15E-04	4.72E-03	**5.95E-01**
**S_2 min (ππ*)**	**11.10 (π^{-1})**	11.21 (n^{-1})	11.77 (π^{-1})	12.04 (n^{-1})
$S_2 \rightarrow D_x$ intensity	**4.23E-01**	1.70E-02	1.68E-02	1.56E-02
**T_1 min (ππ*)**	**10.64 (π^{-1})**	11.16 (n^{-1})	11.79 (π^{-1})	11.82 (π^{-1})
$T_1 \rightarrow D_x$ intensity	**8.13E-01**	6.25E-04	3.49E-03	2.19E-02
**T_2 min (nπ*)**	**10.85 (n^{-1})**	11.00 (π^{-1})	11.95 (π^{-1})	12.27 (n^{-1})
$T_2 \rightarrow D_x$ intensity	**7.95E-01**	3.11E-03	2.03E-04	2.62E-02

Table S3: Excited state energies, vibrational energy gain, ionization energies, and electron binding energy estimated at different geometries of 6AU along the deactivation pathway. Excitation and ionization energies were calculated at the XMS-CASPT2(16,11)/ANO-R2 level of theory using geometries from Borin et al[1]. The binding energies correspond to the sum of ionization energy and vibrational energy gain for the respective states.

Geometry	Excited state energy (eV)	Vibrational energy (eV)	Ionization energy (eV)	Electron binding energy (eV)
**S_0 min**	0.00	0.00	10.43	10.43
**FC point**	5.10	0.00	10.43	10.43
**S_2 (ππ*) min**	4.66	0.44	11.10	11.54
**S_1 (nπ*) min**	4.10	1.00	10.90<br>11.07<br>12.43	11.90<br>12.07<br>13.43
**T_1 (ππ*) min**	3.22	1.88	10.64	12.52
**T_2 (nπ*) min**	4.07	1.03	10.85	11.88

Table S4: The expected shift in the TRPES signal along the electron binding energy axis at different pump wavelengths assuming excitation into the FC region at around 250 nm.

Pump wavelength (nm)	Photon energy (eV)	Expected shift in binding energy (eV)
**284 nm**	4.366	-0.594
**272 nm**	4.558	-0.402
**260 nm**	4.769	-0.191
**250 nm**	4.960	~ 0
**244 nm**	5.081	+0.121

4. Cartesian coordinates

The following xyz geometries, reproduced from Borin et al.,[1] were used for the XMS-CASPT2(16,11)/ANO-R2 calculations in this manuscript.

$S_{0\,min}$ & $S_2(\pi\pi^*)$-FC

C	-5.177890	-0.106410	1.068060
C	-3.758320	-2.473200	0.820420
C	-4.444710	-0.607650	2.245870
N	-5.182760	-0.720420	-0.062050
N	-4.494980	-1.862100	-0.170670
N	-3.771380	-1.788190	2.021480
O	-4.494980	-0.041200	3.303320
O	-3.168020	-3.494580	0.653720
H	-5.733010	0.813710	1.157480
H	-4.526080	-2.308120	-1.064490
H	-3.247540	-2.192830	2.774960

$S_{1\,min}$ $(n\pi^*)$

C	-5.175760	-0.095160	1.094460
C	-3.759870	-2.470530	0.822610
C	-4.479090	-0.613800	2.140450
N	-5.204200	-0.711370	-0.110950
N	-4.491990	-1.865070	-0.168980
N	-3.767630	-1.792470	2.022450
O	-4.408540	-0.044910	3.374690
O	-3.167440	-3.496080	0.660200
H	-5.730080	0.824110	1.184810
H	-4.506260	-2.332480	-1.052320
H	-3.247530	-2.183230	2.780700

$T_{2\,min}(n\pi^*)$

C	-5.168780	-0.089860	1.089600
C	-3.756100	-2.466810	0.822110
C	-4.481040	-0.615650	2.149440
N	-5.205830	-0.710790	-0.106740
N	-4.515690	-1.880750	-0.159740
N	-3.776430	-1.801810	2.026730
O	-4.415860	-0.047680	3.371420
O	-3.136680	-3.473960	0.646430
H	-5.707390	0.839290	1.172810
H	-4.516770	-2.337700	-1.048390
H	-3.257830	-2.195280	2.784430

$S_{2\,min}$ $(\pi\pi^*)$

C	-5.176520	-0.115580	1.092060
C	-3.808230	-2.392200	0.835410
C	-4.477440	-0.544710	2.228380
N	-5.238650	-0.716850	-0.114030
N	-4.481290	-1.874540	-0.164940
N	-3.787960	-1.807330	2.006640
O	-4.353030	-0.051200	3.336580
O	-3.147510	-3.513550	0.644990
H	-5.734160	0.803860	1.168670
H	-4.454940	-2.344140	-1.050500
H	-3.278660	-2.224760	2.764850

$T_{1\,min}$ $(\pi\pi^*)$

C	-5.172590	-0.086010	1.120030
C	-3.766290	-2.456030	0.828690
C	-4.453300	-0.599130	2.236220
N	-5.212860	-0.739050	-0.181240
N	-4.498850	-1.861780	-0.180730
N	-3.767860	-1.791330	2.026050
O	-4.425860	-0.042820	3.321770
O	-3.180820	-3.482090	0.638710
H	-5.726110	0.831310	1.209360
H	-4.487750	-2.350660	-1.050660
H	-3.246100	-2.193400	2.779890

5. Justification of decay model

Initially, the integrated signals were analyzed in Origin with a sequential exponential decay model. For excitation at the shorter pump wavelengths, three exponential functions provide the best fit of the data, but at 272nm and 284nm an extra Gaussian function (G) is needed to account for the initial response. Subsequently, global lifetime analysis was performed in Glotaran 1.5.1[2] to extract spectral and dynamical features. Singular value decomposition (SVD) confirms these initial fitting models. Furthermore, the residual matrix shows only small and random signals attributed to noise. Evolution associated spectra (EAS), and decay rates were determined from the global analysis according to the sequential kinetic model.

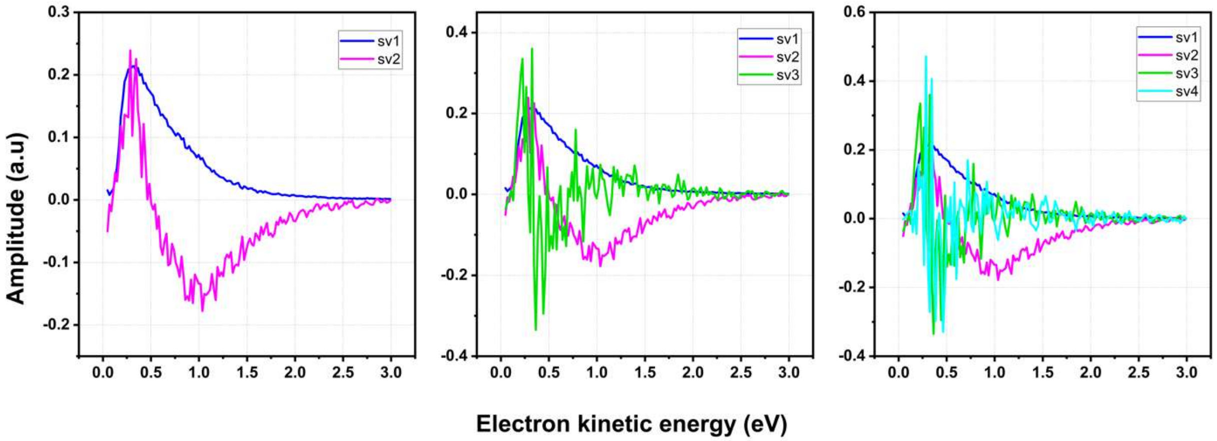

Fig. S2: The singular vectors for the 260 nm dataset, obtained from singular value decomposition analysis, represent the unique components necessary to capture the primary features of the TRPES data. For all pump wavelengths of 243, 250, and 260 nm, three singular vectors are unique while the fourth vector exhibits a similar (but noisier) pattern to the third component and is considered redundant.

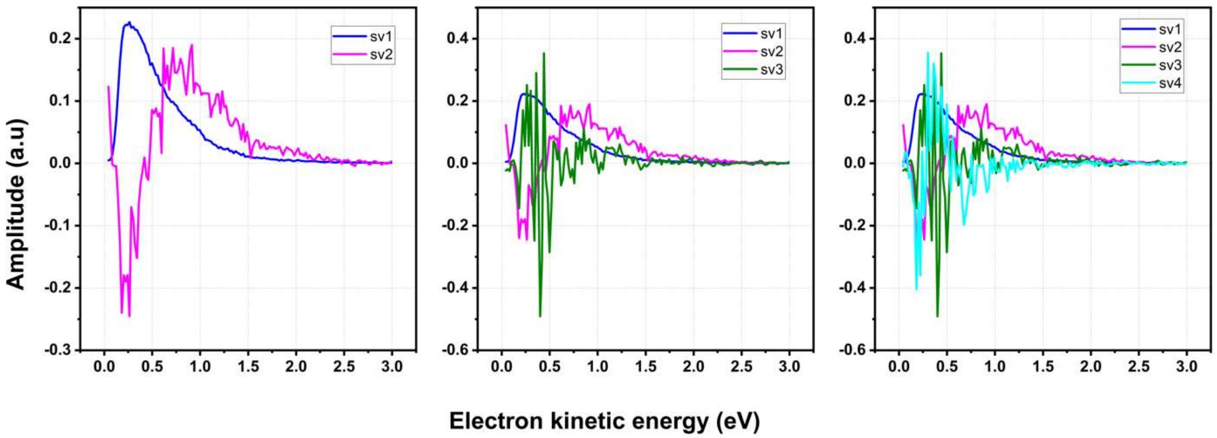

Fig. S3: The singular vectors corresponding to the 284nm dataset reveal four distinct components (one Gaussian and three exponential decays). A similar model is employed for the analysis of the 272nm dataset.

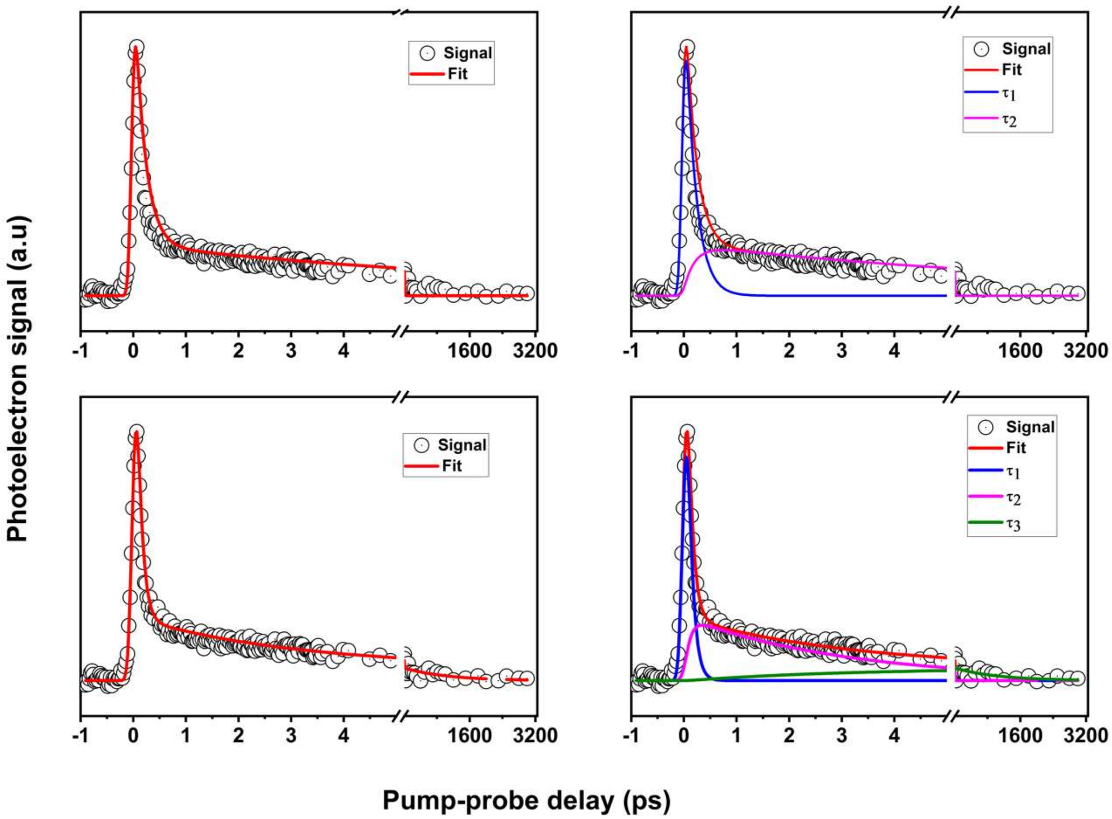

Fig. S4: The fitting strategy is also visually confirmed by fitting the energy integrated signal with a two-decay sequential model (first row) and a three-decay sequential model (second row). For the 260 nm data, the first model is insufficient to obtain a good fit in both the short and the long pump-probe delay range, whereas the inclusion of the third component improves the fit quality. The fits including individual components are shown in the right column.

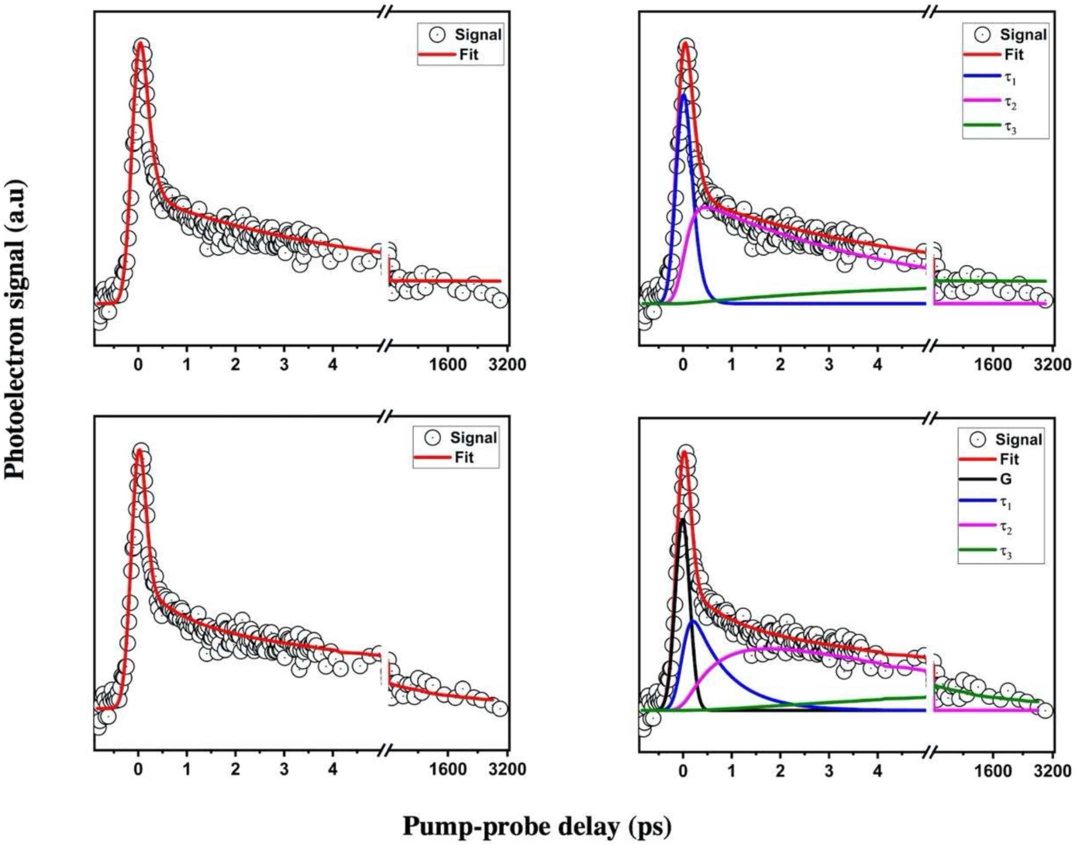

Fig. S5: The fitting strategy for the 284nm data is visualized with fits to the energy integrated signal. A three sequential decay model (first row) is insufficient to capture the signal at both very short and long delays. The figure in the right column includes the total fit and contributions from the three components. The addition of a Gaussian function (second row) significantly improves the fit quality over the entire pump-probe delay range.

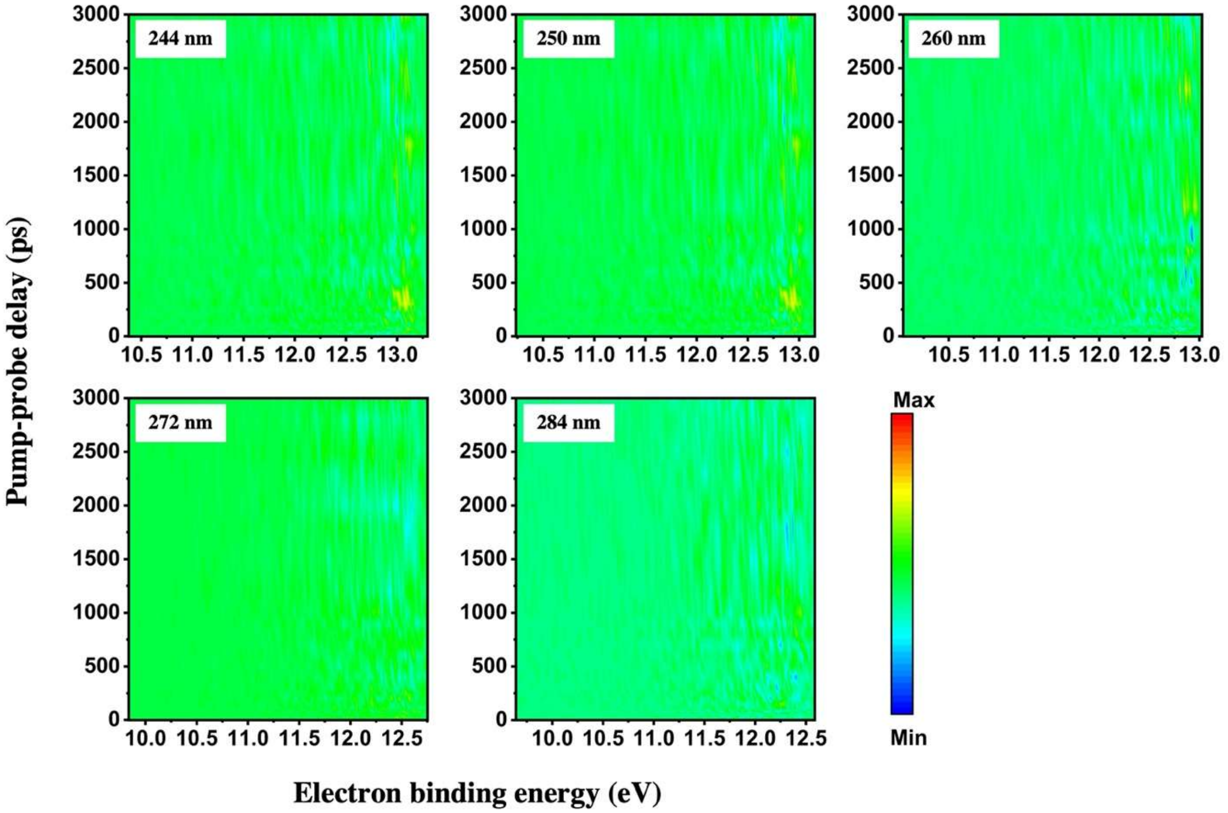

Fig. S6: 2d colormaps of the residuals that remain after global analysis with the sequential decay models discussed above.

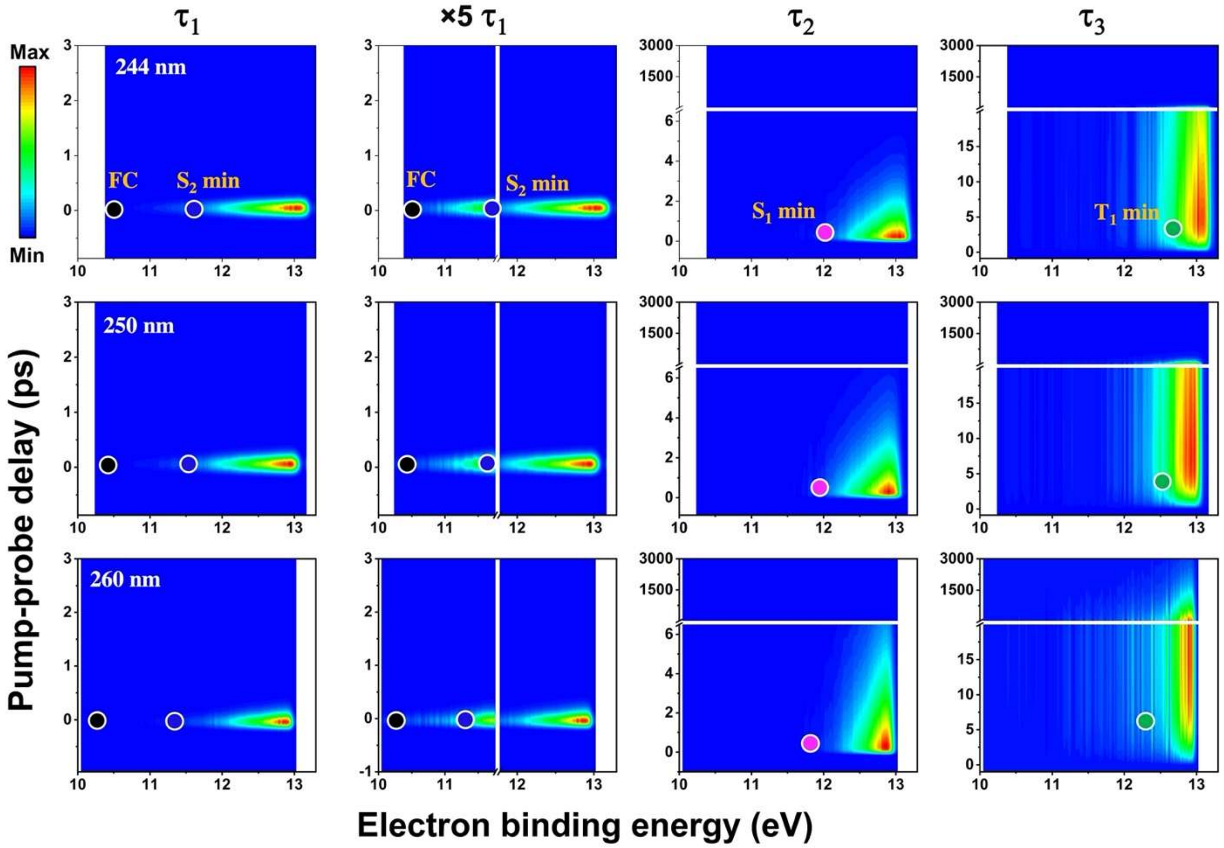

Fig. S7: Colormaps of the three components obtained from the global analysis of the TRPES data. The superimposed colored circles indicate the expected eBE values (from Table S3 but shifted by the values in Table S4) corresponding to the FC region as well as S_2, S_1, and T_1 minima. Each row corresponds to the specified excitation wavelength. Columns 1, 3, and 4 are associated with the timeconstant τ_1, τ_2, and τ_3, respectively. Column 2, is the same as Column 1, i.e., also the component that represents τ_1, but for better visibility of the signal at lower eBEs, the region up to 11.75eV has been multiplied by a factor of 5. In column 2 the signal associated with the ultrafast motion from the FC region to the $S_{2\ min}$ is clearly visible. At these shorter excitation wavelengths, one timeconstant, τ_1, is sufficient to capture the relaxation and population decay dynamics of the S_2 state.

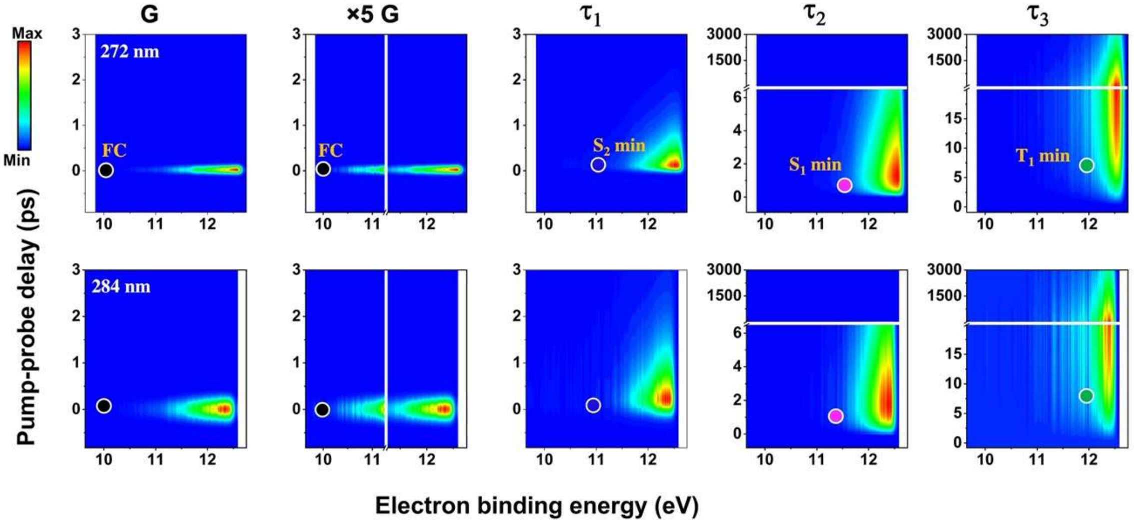

Fig. S8: Colormaps of the four components obtained from the global analysis of the TRPES data. A Gaussian contribution in addition to the three decay constants is required to describe the data. The superimposed colored circles indicate the expected eBE values (from Table S3 but shifted by the values in Table S4) corresponding to the FC region as well as S_2, S_1 and T_1 minima. Each row corresponds to the specified excitation wavelength. Columns 1, 3, 4 and 5 are associated with G, and the timeconstants τ_1, τ_2, and τ_3, respectively. Column 2, is the same as Column 1, i.e., also the component that represents G, but for better visibility of the signal at lower eBEs, the region up to 11.75eV has been multiplied by a factor of 5. In column 2, the signal associated with the ultrafast motion from the FC region to the $S_{2\ min}$ is clearly visible. At these longer excitation wavelengths, two separate timeconstants, G and τ_1, are needed to capture the relaxation and population decay dynamics of the S_2 state, respectively.

Reference

1. Gobbo, J. P.; Borin, A. C.; Serrano-Andrés, L., On the Relaxation Mechanisms of 6-Azauracil. *The Journal of Physical Chemistry B* **2011,** *115* (19), 6243-6251.
2. Snellenburg, J. J.; Laptenok, S.; Seger, R.; Mullen, K. M.; van Stokkum, I. H. M., Glotaran: A Java-Based Graphical User Interface for the R Package TIMP. *Journal of Statistical Software* **2012,** *49* (3), 1 - 22.
